# *Pseudomonas syringae* Lipopolysaccharide Synthesis Gene *wbpL* Displays Heterogeneous Expression Within In Vitro and In Planta Populations

**DOI:** 10.1002/mbo3.70031

**Published:** 2025-07-22

**Authors:** Laura Mancera‐Miranda, José S. Rufián, Nieves López‐Pagán, Javier Ruiz‐Albert, Carmen R. Beuzón

**Affiliations:** ^1^ Dpto. Biología Celular, Genética y Fisiología, Instituto de Hortofruticultura Subtropical y Mediterránea Universidad de Málaga‐Consejo Superior de Investigaciones Científicas (IHSM‐UMA‐CSIC) Málaga Spain

**Keywords:** bacterial gene expression, fluorescence in planta microscopy, fluorescent reporter genes, nongenetic variation, single‐cell methods

## Abstract

Phenotypic heterogeneity usually refers to phenotypic variation not associated with genetic variation, nor induced by environmental stimuli. The phenotypic heterogeneity processes described for some complex bacterial traits are causing a shift in how bacterial phenotypes are studied, from traditional assessments by averaging populations to single‐cell analysis focused on bacterial individual phenotypes and how these distribute within the population. The structure of the lipopolysaccharide (LPS) layer on the outer membrane in Gram‐negative bacteria is often subjected to phenotypic variation, a process that is critical for virulence in animal pathogens. Here, we apply single‐cell expression analyses to *wbpL*, a conserved *Pseudomonas syringae* glycosyltransferase‐encoding gene essential for the synthesis of the *O*‐antigen component of LPS. We show that expression of *wbpL* displays phenotypic heterogeneity in *P. syringae* pv. *phaseolicola* growing in rich medium and reaches bistable expression in minimal medium, where the population splits into WbpL^ON^ and WpbL^OFF^ subpopulations. In planta, *wbpL* expression is also heterogeneous, displaying intermingled ON/OFF with comparable viability. Finally, we followed the expression of *wbpL* within the spatial context of apoplastic microcolonies, and not only detected heterogeneity within each microcolony, but also found that microcolonies displayed overall differences in fluorescence intensity that correlated with size, with smaller microcolonies displaying higher levels of *wbpL* expression.

## Graphical abstract

Genetically identical bacteria in the same environment sometimes behave differently. This happens for the expression of *wbpL*, a gene of plant pathogenic *Pseudomonas syringae* that shapes the bacterial surface and is important for infection and immunity. whose expression varies among individual bacteria, with ON/OFF bacteria coexisting with similar viability. In planta, the smaller size of microcolonies with higher *wbpL* expression suggests *wbpL* expression might slow growth or trigger stronger defenses. Phenotypic heterogeneity of lipopolysaccharide genes is a common strategy for plant and animal pathogens.

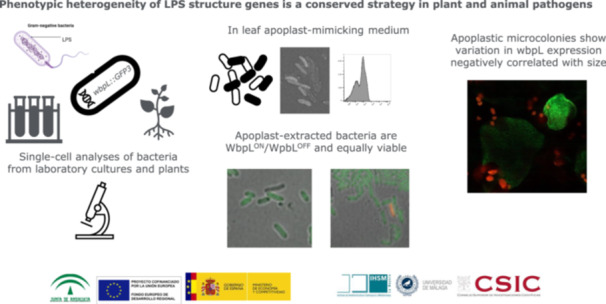

## 1 Introduction

The outer membrane (OM) is a hallmark of Gram‐negative bacteria that is essential for bacterial viability (Silhavy et al. 2010). The OM is an atypical membrane formed by an asymmetric lipid bilayer, with phospholipids in the inner side and lipopolysaccharides (LPSs) in the outer (Funahara and Nikaido 1980). LPSs are a family of structurally related glycolipids that contain a mix of well‐conserved and highly variable structural elements. LPS is formed by three different and covalently linked domains (Whitfield et al. 2020): (i) a conserved lipophilic lipid A moiety, with a major role in permeability, which is recognized by the innate immune system as a pathogen‐associated molecular pattern in host–microbe interactions (Whitfield et al. 2020); (ii) a short hydrophilic core oligosaccharide structure (core OS), which contributes to OM stability (Holst and Brade 1992); and in most cases (iii) a long‐chain hypervariable repeat‐unit *O*‐polysaccharide (OPS) also known as *O*‐antigen that glycosylates core OS, and may differ in glycose and nonglycose components, linkages, topology and chain lengths (Goldman and Leive 1980). The OPS is often involved in providing protection against environmental threats (Whitfield et al. 2020), phage recognition and binding (Mostowy and Holt 2018), in adhesion to surfaces and biofilm formation (Bogino et al. 2013), and as the exposed part of LPS, the OPS can also contribute to the recognition of LPS by the immune system in both animal and plant hosts (Lerouge and Vanderleyden 2002; Ranf 2016).

Phenotypic variation of OPS is a critical aspect of virulence in many Gram‐negative animal pathogens, for example, *Coxiella burnetii* (Long 2024), *Helicobacter pylori* (Appelmelk et al. 2000; Sijmons et al. 2022), *Haemophilus influenzae* (Weiser et al. 1990), *Pasteurella multocida* (Omaleki et al. 2022), *Francisella tularensis* (Mlynek et al. 2022), or *Salmonella enterica* (Cota et al. 2012). No such processes have been reported for OPS in plant pathogens. Nonetheless, defects in OPS synthesis have been shown to cause a complete or strong virulence attenuation in plant pathogens, such as *Erwinia* spp., *Ralstonia solanacearum, Xanthomonas axonopodis* pv. *citri* or *Xylella fastidiosa* (Drigues et al. 1985; Schoonejans et al. 1987; Berry et al. 2009; Petrocelli et al. 2012; Li et al. 2014; Rapicavoli et al. 2018). In the model plant pathogen *Pseudomonas syringae* species complex, a genetic analysis identified *wbpL*, a gene encoding a glycosyltransferase, as conserved in all strains examined and essential for OPS synthesis (Kutschera et al. 2019). Deletion of *wbpL* in the model strain *P. syringae* pv. tomato DC3000 reduces the ability of this pathogen to enter the host tissue. This is likely associated with the reduced swarming motility displayed by the Δ*wbpL* mutants in this pathogen (Kutschera et al. 2019). The Δ*wbpL* mutant also has a reduced ability to colonize the leaf apoplast of host plant *Arabidopsis thaliana* (Kutschera et al. 2019). Mutations of the *wbpL* ortholog of another model strain from this species complex, *P. syringae* pv. *phaseolicola* 1448A had also been reported previously to reduce leaf colonization and virulence in common bean (Rudolph 1989).

In this study, we have investigated the expression of *wbpL* at the single‐cell level within populations of two important *P. syringae* model strains where this gene has been proven to be involved in virulence: *P. syringae* pv. tomato DC3000 (hereafter Pto) and *P. syringae* pv. *phaseolicola* 1448A (hereafter Pph). We have generated transcriptional fusions to a promoterless green‐fluorescent protein 3 (*GFP3*) gene downstream to the *wbpL* gene of each of these two model strains and used single‐cell methods to follow the expression of this gene both under laboratory growing conditions and in plants. We show that expression of *wbpL* is low and displays phenotypic heterogeneity in *P. syringae* pv. *phaseolicola* growing in rich medium, while it reaches bistable expression in minimal media, where the population splits into WbpL^ON^ and WpbL^OFF^ subpopulations. In planta, *wbpL* expression is also heterogeneous, with the apoplastic population displaying intermingled ON/OFF bacteria with comparable viability. Finally, we followed the expression of *wbpL* within the spatial context of apoplastic microcolonies, detecting heterogeneity within each microcolony. In this context, we also found that microcolonies displayed overall differences in fluorescence intensity that negatively correlated with size, with smaller microcolonies displaying higher levels of *wbpL* expression.

## 2 Materials and Methods

### 2.1 Bacterial Strains and Growth Conditions

Bacterial strains used and generated in this study are detailed in Table 1. *Escherichia coli* and *P. syringae* strains were grown with aeration in Lysogeny Broth (LB) medium (Bertani 1951) at 37°C (*E. coli*) or 28°C (*P. syringae*). When necessary, antibiotics were used at the following concentration: ampicillin (Amp), 100 μg/mL for *E. coli* and 500 μg/mL for *P. syringae*; kanamycin (Km), 50 μg/mL for *E. coli* and 15 μg/mL for *P. syringae* derivative strains; cycloheximide, 2 μg/mL. We also used Hrp‐inducing medium (HIM), which is believed to mimic conditions within the leaf apoplast. Different studies have used variations on the composition of this minimal medium (Bretz et al. 2002; Huynh et al. 1989; Rahme et al. 1992; Taira et al. 1999; Thwaites et al. 2004; Xiao et al. 1992). The formulation and pH used in this study (Xiao et al. 1992) provide equal results in single‐cell type III secretion system (T3SS) expression analysis regardless of pH (pH 5.7 or 7.0), fitting with T3SS expression throughout pH‐changing early stages of apoplast colonization (Rufián et al. 2016; O'Leary et al. 2016; López‐Pagán et al. 2025). To grow cultures in HIM, bacteria were initially cultured overnight in LB at 28°C, supplemented with the appropriate antibiotic, then washed twice in 10 mM MgCl_2_ and then grown from an initial optical density (OD) adjusted to 0.13 in hrp‐inducing minimal medium (HIM), containing 10 mM fructose, and pH adjusted to 7 with 10 N NaOH, at 28°C with agitation.

**Table 1:** List of bacterial strains used in this study.

Name	Genotype	Reference
*Escherichia coli* One Shot TOP10	*F‐mcrA Δ(mrr‐hsdRMS‐mcrBC) Φ80lacZΔM15 ΔlacX74 recA1 araD139 Δ(araleu)7697 galU galK rpsL (StrR) endA1 nupG*. For cloning	Invitrogen
1448A	*Pseudomonas syringae* pv. *phaseolicola* wild‐type strain race 6	Teverson (1991)
DC3000	*P. syringae* pv. tomato wild‐type strain	Cuppels (1986)
LMM1	1448A *wbpL::GFP3* Km^R^	This study
LMM2	DC3000 *wbpL::GFP3* Km^R^	This study

### 2.2 Generation of Strains Carrying a *wbpL::GFP3* Transcriptional Fusion

Bacterial strains carrying a chromosome‐located transcriptional fusion of *wbpL* to a promoterless *GFP3* gene were generated using an adaptation of the method previously described by Rufián. (2018). The plasmids used and generated for this purpose are detailed in Table 2, and the primers used are described in Table 3. To generate the allelic exchange plasmids, we amplified two fragments from either Pph 1448A or Pto DC3000 genomic DNA using Q5 High‐Fidelity DNA Polymerase (New England Biolabs, USA); in each case, fragment A encompasses the 3′ end of the *wbpL* open reading frame (ORF), including the STOP codon, and fragment B the sequence immediately downstream to the STOP codon. Reactions were carried out starting at 98°C for 1 min for the initial denaturation step, followed by 30 cycles at 98°C for 30 s, annealing at 58°C (fragment B) or 60°C (fragment A) for 30 s, and extension at 72°C for 30 s, followed by 5 min at 72°C for the final extension step. Reaction mixes included 0.64 mM deoxynucleotide triphosphate (dNTP) mix, 0.4 ng of each primer, 1 ng of genomic DNA, the appropriate enzyme buffer, and commercial ultrapure water (Nalgene, Rochester, NY, USA). Two μL of each gel‐purified product of the polymerase chain reaction (PCR) was employed as templates for the subsequent fusion PCRs, employing primers A1 and B2, for reactions carried out as described above, with extended elongation times of 1 min. The resulting bands, containing the end of each ORF and its downstream sequence separated by an EcoRI restriction site, were cloned into comercial plasmid pGEM‐T (Promega, USA) and fully sequenced to disregard mutations. This process rendered the pGT‐AB‐*wbpL* plasmids needed for generating the allelic exchange plasmids. A *GFP3*‐FRT‐*nptII*‐FRT fragment was obtained by digesting the plasmid pGT‐GFP with the EcoRI enzyme. This fragment includes the promoterless *GFP3* gene, with its ribosomal binding site, followed by the kanamycin resistance gene (*nptII*), flanked by flipase recognition targets (FRTs) sites, and is flanked by two EcoRI restriction sites. The *GFP3*‐FRT‐*nptII*‐FRT fragment was blunt‐ended through a PCR procedure and ligated into EcoRI‐digested pGT‐AB‐*wbpL* plasmids, generating the pGT‐*wbpL::GFP3* plasmids. These plasmids were transformed into the corresponding wild‐type strains, *P. syringae* pv. *phaseolicola* 1448A to generate the Pph *wbpL::GFP3* strain, and *P. syringae* pv. tomato DC3000 to generate the Pto *wbpL::GFP3* strain.

**Table 2:** List of plasmids used and generated in this study.

Name	Description	Reference
pGEM‐T vector	*Escherichia coli* expression vector for cloning. Amp^R^	Promega, USA
pGT‐GFP^+^	*E. coli* expression vector carrying the *GFP3‐Km* gene. Amp^R^ Km^R^	Rufián (2018)
pGT‐AB‐Pto‐*wbpL*	pGEM‐T vector carrying the last 600 pb of the *wbpL* ORF from DC3000 (A) followed by an EcoRI site and the 500 bp immediately downstream the *wbpL* STOP codon (B). Amp^R^	This study
pGT‐Pto‐*wbpL*::*GFP3*	Allelic exchange vector for the generation of a transcriptional fusion of *wbpL* to *GFP3* in DC3000. Amp^R^ Km^R^	This study
pGT‐AB‐Pph‐*wbpL*	pGEM‐T vector carrying the last 600 pb of the *wbpL* ORF from 1448A (A) followed by an EcoRI site and the 600 bp immediately downstream the *wbpL* STOP codon (B). Amp^R^	This study
pGT‐Pph‐*wbpL*::*GFP3*	Allelic exchange vector for the generation of a transcriptional fusion of *wbpL* to *GFP3* in 1448A. Amp^R^ Km^R^	This study

**Table 3:** List of primers used in this study.

Name	Sequence	Restriction site	Reference
A1 *PphwbpL*	CTGGTATGGATGTTGAATCT	—	This study
A2 *PphwbpL*	CCGCTTCGGAATTCTTACCTGATTTCCGCCT	*EcoRI*	This study
B1 *PphwbpL*	TCAGGTAAGAATTCCGAAGCGGTTTTCTG	*EcoRI*	This study
B2 *PphwbpL*	TAAATGGCGACCTTCTG	—	This study
A1 *PtowbpL*	CTGGTATGGATGTTGAATCT	—	This study
A2 *PtowbpL*	CCGCTTCGGAATTCTTACCTGCTTTCTGCCT	*EcoRI*	This study
B1 *PtowbpL*	GCAGGTAAGAATTCCGAAGCGGGTTTCTG	*EcoRI*	This study
B2 *PtowbpL*	TAGATGGCGACTTTTTGC	—	This study

### 2.3 Plant Assays

Ten‐day‐old *Phaseolus vulgaris* bean cultivar Canadian Wonder plants cultivated under controlled conditions at 23°C, 95% humidity, with artificial light maintained for periods of 16 h within the 24 h were used in all experiments. The first true leaves were inoculated by immersing the entire leaf in the bacterial inoculum, containing 0.01% Silwett L‐77 (Crompton Europe Ltd., Evesham, UK), and using a pressure chamber (Rufián et al. 2022). Bacterial inocula were prepared resuspending biomass from bacterial lawns grown on LB plates for 48 h at 28°C, into 2 mL of 10 mM MgCl_2_, adjusting optical density (OD600) to 0.1 (which corresponds to 5 × 107 colony forming units per milliliter, CFU/mL, of Pph 1448A), and serially diluting to a final concentration of 5 × 104 CFU/mL. Four days postinoculation (dpi), bacteria were recovered from the plant apoplastic fluid as previously described (Rufián et al. 2022). Briefly, each leaf is pressure infiltrated with 10 mL of a 10‐mM MgCl_2_ solution inside a 50‐mL syringe, by applying five cycles of pressure (pushing and pulling the plunger five times), before collecting the flow‐through, which is then transferred to a fresh 50‐mL tube from which 3 mL are directly analyzed by flow‐cytometry (FC).

### 2.4 FC Analysis

LB cultures were obtained from an overnight incubation in LB. For HIM cultures, 500 μL of an overnight *P. syringae* LB culture was washed twice in 10 mM MgCl_2_, added to 4.5 mL of HIM and incubated at 28°C for 24 h (Pph) or 5 h (Pto). Apoplast‐extracted bacterial suspensions were obtained as indicated above. Three hundred μL of the cultures in HIM, LB or apoplast‐extracts were analyzed using a BD FACS Verse cytometer (BD Biosciences, USA). FITC‐A filter was used to visualize green fluorescent protein (GFP) signal. Graphs were generated using the FlowJo X v. 10.0.7r software.

### 2.5 Confocal Microscopy Analysis

Suspensions of 2 μL of apoplast‐extracted bacteria or media‐grown bacteria were deposited over a 0.17‐mm coverslip and an agar‐pad square was placed on top of the drop to create a bacterial monolayer, following the method described in Rufián et al. (2022). To visualize all cells, bright field images were included. Images of single‐cell bacteria were acquired using the Zeiss LSM880 confocal microscope (Zeiss, Germany), using ×63 or ×100 objectives. To visualize apoplastic microcolonies, sections of *P. vulgaris* leaves (approximately 5 mm^2^) inoculated with Pph *wbpL::GFP3*, were carefully excised using a razor blade, mounted on slides in double‐distilled H_2_O with the lower epidermis facing the objective, and cover with a 0.17‐mm coverslip. Images of the leaf mesophyll were taken using the Leica Stellaris 8 confocal microscope (Leica Microsystems GmbH, Germany) with ×40 objectives. Filters for wavelength selection were used for the visualization GFP (488 nm/500–533 nm), and plant autofluorescence (514/605–670 nm). Images were processed using Leica LASX 1.4.6 (Leica Microsystems, Germany) software. Z series imaging was taken at 1 μm using ×40 objectives.

### 2.6 Live‐Dead Stanning

We added one drop of a propidium iodide solution Ready Probes (Thermo Fisher Scientific, USA) to 300 μL of the suspension of apoplast‐extracted bacteria. Live‐dead bacteria were identified by FC. For live‐dead staining, bacteria were syringae‐infiltrated with a suspension of 5 × 104 CFU/mL in bean leaves and apoplast‐extracted at 4 dpi. Live‐dead bacteria were identified according to PI staining by confocal microscopy (as described above).

### 2.7 Quantification and Statistical Analysis

Quantification and statistical analysis were performed using Prism. Details of the analysis used and the level of significance are indicated in the corresponding figure legends. Software used for data quantification and analysis is detailed in Table 4.

**Table 4:** List of software used in this study.

Software	Reference	Source
Image J 2.9.0/Fiji 2.14.0		https://imagej.net/ij/docs/index.html
GraphPad Prism 9.0	Prism	https://www.graphpad.com
LAS X 1.4.6	Leica microsystem	https://www.leica-microsystems.com/es/productos/software-de-microscopia/p/leica-las-x-ls/
ZEN 3.4	Carl Zeiss Microscopy	https://www.zeiss.com/microscopy/es/productos/software/zeiss-zen.html
BDFACSDiva	BD	https://www.bdbiosciences.com/ko-kr/products/software/instrument-software/bd-facsdiva-software
BDFACSuite 1.0.5	BD	https://www.bdbiosciences.com/en-ie/products/software/instrument-software/bd-facsuite-application#Overview
FlowJo X v. 10.0.7r	Tree Star	https://www.flowjo.com

## 3 Results

### 3.1 Single‐Cell Expression Analysis of *wbpL* in Pph 1448A in Laboratory Conditions

**Figure 1:**
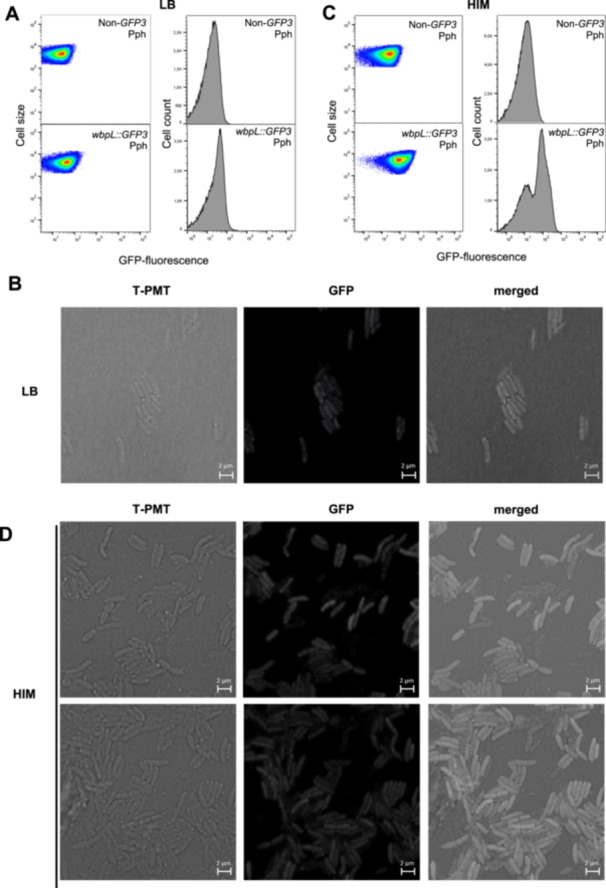
*Pseudomonas syringae* pv. *phaseolicola* 1448A *wbpL* displays phenotypic heterogeneity in LB‐grown populations and bistability in HIM‐grown. (A, C) Flow‐cytometry analysis of a Pph derivative strain carrying a chromosome‐located *wbpL*:*:GFP3*, grown 24 h in LB (A) or in HIM (C) is shown as dot plots representing GFP fluorescence intensity versus cell size. Data are represented as arbitrary units on a logarithmic scale. Histograms show GFP fluorescence versus cell count for the same data. Data displayed corresponds to that collected for at least 100,000 events per sample. The non‐GFP graphs show autofluorescence levels displayed by the Pph reference strain not carrying any fluorescent gene marker. Confocal microscopic images LB‐grown (B) or HIM‐grown (D) of Pph *wbpL*:*:GFP3*. Microscopy images show in the GFP channel the fluorescence of GFP (in white) as a reporter of *wbpL* gene expression. Bright field is used to visualize all bacteria regardless of *wbpL* expression (merged). Scale bars correspond to the values indicated. Contrast and brightness were adjusted to improve visualization, but were kept constant across the different conditions and channels. Microscopy and cytometry panels show typical results of at least three independent replicates. GFP, green fluorescent protein; HIM, Hrp‐inducing medium; LB, Lysogeny Broth.

To investigate single‐cell expression of *wbpL* within populations of Pph (1448A), we generated a transcriptional fusion to a promoterless *GFP3* gene downstream to the *wbpL* (PSPPH_3657) gene within its native chromosome location (Figure S1). This was carried out following previously described methodology based on allelic exchange that preserves genome context and the target gene sequence (Rufián 2018; Figure S1). Cultures grown in rich medium (LB) of the resulting Pph *wbpL::GFP3*, as well as of the corresponding Pph reference strain, were used for single‐cell expression analysis. FC shows that *wbpL* is expressed at a very low level, and not for the entire population (Figure 1A), with just approximately 10% of WbpL^ON^ bacteria in Pph *wbpL::GFP3* populations detected by FC on average. FC histograms consistently show a unimodal distribution for *wbpL::GFP3* fluorescence that considerably overlaps with the corresponding nonfluorescent Pph reference strain used as a negative control (Figure 1A). This fluorescence distribution was maintained regardless of the growth phase (Figures 1A and S2). Confocal microscopy analysis of GFP fluorescence within Pph *wbpL::GFP3* populations shows a few bacteria displaying very low, but higher than most, fluorescence intensities (hereafter referred to as WbpL^ON^ bacteria, in reference to their expression phenotype) (Figure 1B), in keeping with FC results (Figure 1A). Control images obtained for the non‐*GFP3* Pph reference strain taken with these settings show that the very low background expression that can be detected in some Pph *wbpL::GFP3* is due to expression of the gene fusion and not to background noise (Figure S3).

We also analyzed the single‐cell expression of *wbpL::GFP3* in bacterial populations grown in a minimal medium (Hrp‐inducing medium or HIM) where expression of the T3SS genes is induced and that is believed to mimic to some extent conditions within the plant apoplast (Huynh et al. 1989; Xiao et al. 1992). FC analysis carried out on HIM‐grown populations of Pph *wbpL::GFP3* and Pph show results rather different to those previously observed for LB‐grown (Figure 1C). First, the percentages of WbpL^ON^ bacteria within the HIM‐grown populations were consistently higher than those observed in those LB‐grown (Figure 1C), with half of the bacteria, on average, displaying a stronger WbpL^ON^ phenotype. This change in fluorescence distribution is accompanied by a significantly larger variation in fluorescence levels among HIM growing bacteria than those detected among those LB‐grown (as established by a robust coefficient of variation or RCV, Figure S2). Second, histograms for these samples reproducibly show a marked bimodal (sometimes even trimodal) distribution of fluorescence (Figure 1C). This distribution is compatible with a bistable expression pattern and the formation of WbpL^ON^ and WbpL^OFF^ subpopulations, the latter fully overlapping with the nonfluorescent reference Pph strain (Figure 1C). The term bistability is used to describe bimodal patterns of gene expression. These patterns can be determined by phase variation (van der Woude 2011), as in the case of *opvAB*, which determines the length of the *O*‐antigen in the LPS of *S. enterica* (Cota et al. 2012), but also when loci with heterogeneous (noisy) expression are regulated by positive feedback loops, as in the paradigmatic case of the *E. coli lac* operon (Novick and Weiner 1957), or double negative feedback loops as in another classic example of regulation, the lysis/lysogeny decision in the λ bacteriophage (Herskowitz and Hagen 1980). Such regulatory loops split heterogeneous populations into ON/OFF subpopulations. Confocal microscopy analysis supports the results obtained by FC (Figure 1D). Although the fluorescence intensities displayed by WbpL^ON^ bacteria in HIM‐grown populations are not high, they are distinctly higher than in LB, to such extend that WbpL^ON^ and WbpL^OFF^ bacteria can be easily identified within these populations (Figure 1D). Our team has previously reported bistable expression for T3SS (Hrp) genes in HIM‐grown populations of Pph (Rufián et al. 2016). In the case of the T3SS genes, bistability is linked to a double‐negative feedback loop, and enhanced by an additional positive feedback loop (Rufián et al. 2016).

### 3.2 Single‐Cell Expression Analysis of *wbpL* in Pto DC3000 in Laboratory Conditions

**Figure 2:**
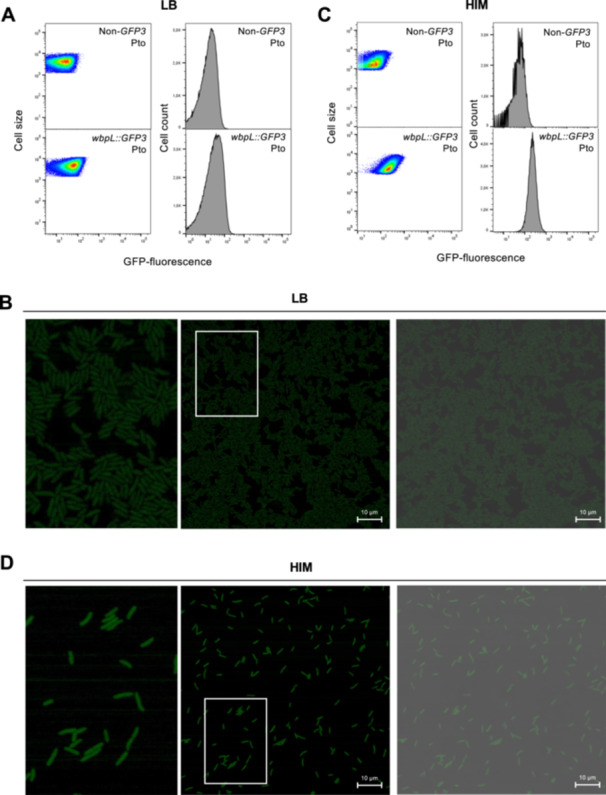
*Pseudomonas syringae* pv. *tomato* DC3000 *wbpL* does not display phenotypic heterogeneity in LB‐grown populations nor bistability in HIM‐grown. (A, C) Flow‐cytometry analysis of a Pto derivative strain carrying a chromosome‐located *wbpL*:*:GFP3*, grown 24 h in LB (A) or 5 h in HIM (C) is shown as dot plots representing GFP fluorescence intensity versus cell size. Data are represented as arbitrary units on a logarithmic scale. Histograms show GFP fluorescence versus cell count for the same data. The data displayed correspond to that collected for at least 100,000 events per sample. The non‐GFP graphs show autofluorescence levels displayed by the Pto reference strain, not carrying any fluorescent gene marker. Confocal microscopic images LB‐grown (B) or HIM‐grown (D) of Pto *wbpL*:*:GFP3*. Microscopy images show in the GFP channel the fluorescence of GFP (in white) as a reporter of *wbpL* gene expression. The bright field is used to visualize all bacteria regardless of *wbpL* expression (merged). Scale bars correspond to the values indicated. Contrast and brightness were adjusted to improve visualization but were kept constant across the different conditions and channels. Microscopy and cytometry panels show typical results of at least three independent replicates. GFP, green fluorescent protein; HIM, Hrp‐inducing medium; LB, Lysogeny Broth.

To investigate the expression of *wbpL* at the single‐cell level within populations of Pto (DC3000), we generated transcriptional fusions to a promoterless *GFP3* gene downstream to the *wbpL* (PSPTO_1755) gene within its native chromosome location, as carried out for Pph (Figure 1) to preserve genome context and the target gene sequence (Rufián 2018). Cultures grown in rich medium (LB) of the resulting Pph *wbpL::GFP3*, as well as of the corresponding Pph reference strain, were used for single‐cell expression analysis. FC and confocal analysis carried out using LB‐grown Pto *wbpL::GFP3* cultures (Figure 2A,B) show fluorescence levels were very low and arguably less heterogeneous than observed for Pph *wbpL::GFP3* (Figure 1A,B). Histograms show unimodal distributions for Pto *wbpL::GFP3* fluorescence extensively overlapping with the corresponding nonfluorescent Pto reference strain (Figure 2A), supporting what can be observed by confocal microscopy (Figure 2B). FC analyses carried out with 5 h HIM‐grown Pto *wbpL::GFP3* cultures, using shorter incubation times to account for the faster growth rate of Pto versus Pph in this medium, we also found a unimodal distribution of fluorescence (Figure 2C), clearly differing from the bimodal distribution displayed by Pph *wbpL::GFP3* in HIM (Figure 1C). Also, although expression is slightly induced in HIM versus LB‐grown Pto *wbpL::GFP3*, it is lower on average than expression displayed by Pph *wbpL::GFP3* WbpL^ON^ bacteria (Figure 2C vs. Figure 1C). Confocal microscopy analysis showed little differences in the GFP fluorescence displayed by individual bacteria (Figure 2D), contrasting with the differences observed between WbpL^ON^ and WbpL^OFF^ in HIM‐grown Pph *wbpL::GFP3* bacteria (Figure 1D). Therefore, although we cannot rule out that the Pto *wbpL* locus may display heterogeneity or even bistable expression in a different environment, our results do not support this being the case under the conditions used in this study. Thus, from this point onwards, we focused our study on the Pph *wbpL* locus.

### 3.3 Pph Heterogeneously Expresses *wbpL* Within the Plant Leaf Apoplast

**Figure 3:**
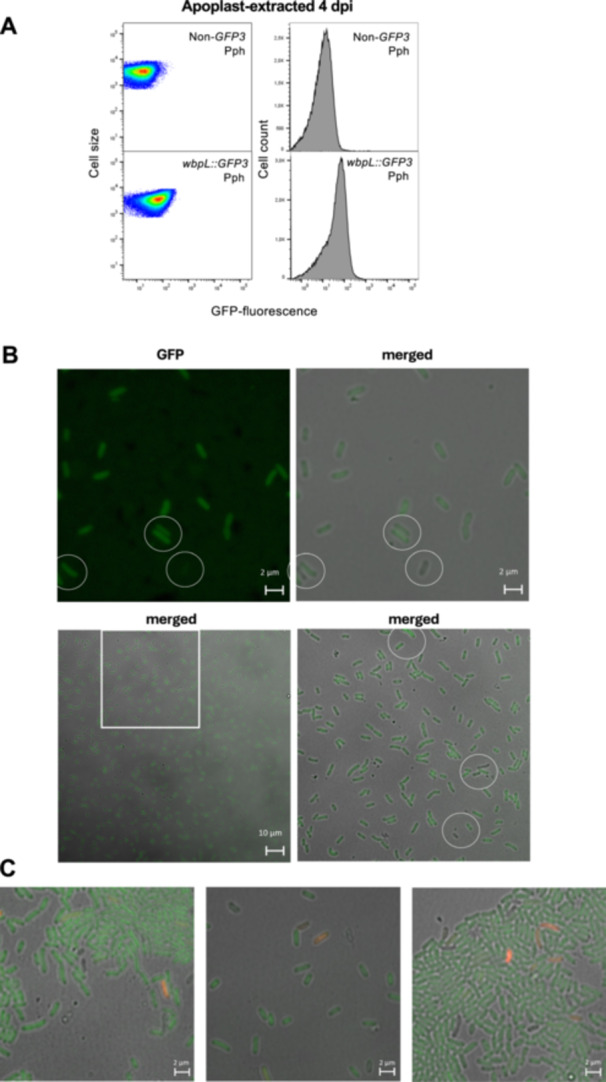
Expression of Pph *wbpL::GFP3* is heterogeneous in apoplast‐extracted bacteria. (A) Flow‐cytometry analysis of Pph *wbpL*:*:GFP3* extracted from the apoplast of 4 days postinoculation bean leaves shown as dot plots representing GFP fluorescence intensity versus cell size. Data are represented as arbitrary units on a logarithmic scale. Histograms show GFP fluorescence versus cell count for the same data. The data displayed correspond to that collected for at least 100,000 events per sample. The non‐GFP graphs show autofluorescence levels displayed by the Pph reference strain, not carrying any fluorescent gene marker. (B) Selected images of apoplast‐extracted Pph *wbpL*:*:GFP3* bacteria. Confocal microscopic images show in the GFP channel the fluorescence of GFP as a reporter of *wbpL* gene expression. The bright field is used to visualize all bacteria regardless of *wbpL* expression. Scale bars correspond to the values indicated. Contrast and brightness were adjusted to improve visualization but were kept constant across the different conditions and channels. Microscopy and cytometry panels show typical results of at least three independent replicates. (C) Selected images of apoplast‐extracted Pph *wbpL*:*:GFP3* bacteria stained with propidium iodide (red). Confocal microscopic images show in the GFP channel the fluorescence of GFP as a reporter of *wbpL* gene expression. The bright field is used to visualize all bacteria regardless of *wbpL* expression. Scale bars correspond to the values indicated.

Although the *wbpL* locus is heterogeneously expressed in both LB and HIM in Pph *wbpL::GFP3*, the formation of ON/OFF subpopulations is only detected in HIM‐grown populations. Since HIM conditions are closer to conditions within the leaf apoplast, we next evaluated *wbpL::GFP3* expression in populations grown in planta, where the function of *wbpL* has been shown to be involved in virulence. To do this, we inoculated *P. vulgaris* (common bean) leaves and bacteria were recovered from the plant apoplastic fluid, as previously described (Rufián et al. 2022), 4 dpi. FC analyses of apoplast‐extracted Pph *wbpL::GFP3* bacteria show a heterogeneous expression pattern for this locus following colonization of the bean leaf (Figure 3A). Confocal microscopy analysis supports the heterogeneous activation of the locus as shown by FC, with WbpL^OFF^ bacteria readily identified among apoplast‐extracted bacteria (Figure 3B). The T3SS genes in Pph, which display bistable expression during growth in HIM, also display heterogeneous expression during growth in the more complex plant environment (Rufián et al. 2016).

In *Arabidopsis*, the receptor kinase LIPOOLIGOSACCHARIDE‐SPECIFIC REDUCED ELICITATION/S‐DOMAIN‐1‐29 (LORE/SD1‐29) mediates medium‐chain 3‐hydroxy fatty acid (mc‐3‐OH‐FA) perception only in their free form, since 3‐OH‐decanoyl‐comprising bacterial compounds, like, the lipid A from LPS, do not stimulate LORE‐dependent responses (Ranf et al. 2015; Kutschera et al. 2019). There are many potential sources of free mc‐3‐OH‐FA in bacteria (discussed in Kutschera et al. 2019). It is conceivable that the absence or presence of properly formed *O*‐antigen may affect lipid A exposure, altering the release of mc‐3‐OH‐FA derivatives susceptible to stimulate LORE‐dependent signaling. In addition, the OPS is often involved in providing protection against environmental threats that could include plant defense compounds, bacteriocins from competing bacteria or bacteriophages (Whitfield et al. 2020). Therefore, differences in expression in *wbpL* within the apoplast could potentially have an impact on how each individual bacteria is detected by plant immune receptors and/or its ability to withstand these defenses and/or additional environmental challenges encountered within the apoplast, since mutations in LPS can be pleiotropic. To evaluate whether any of such processes have a differential impact on WbpL^ON^ or WbpL^OFF^ bacterial viability, we used propidium iodide (PI) to detect membrane‐compromised (not viable) apoplast‐extracted bacteria. This approach can help in determining whether any of the phenotypic variants is undergoing higher killing rates during colonization of the apoplast (Lehtinen et al. 2004; Patel et al. 2021). We found no preferential association of PI staining with WbpL^ON^ or WpbL^OFF^ bacteria within the apoplast‐extracted populations (Figure 3C). A similar approach has been applied previously to evaluate killing rates within the apoplast of T3SS^ON^/T3SS^OFF^ and Flagella^ON^/Flagella^OFF^, which also displays heterogeneous expression in this niche, revealing no significant differences between the dead/live ratios of ON versus OFF subpopulations for any of the T3SS/Flagella phenotypic variants (López‐Pagán et al. 2025).

### 3.4 Phenotypic Heterogeneity Between Apoplastic Microcolonies

**Figure 4:**
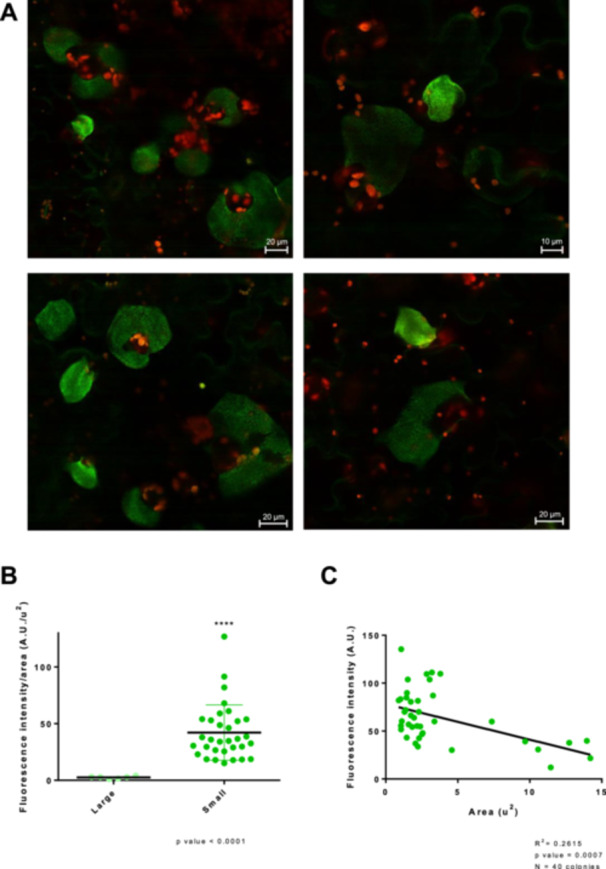
Expression of *wbpL::GFP3* within the apoplast displays stochastic variation and a negative correlation with the size of the microcolonies. (A) Selected images of apoplastic microcolonies of Pph *wbpL*::*GFP3* taken 4 days postinoculation (dpi) of bean leaves with 5 × 10^4^ CFU/mL bacterial suspensions. Heterogeneity is displayed throughout the microcolony. A Stellaris 8 microscope (Leiva Microsystems) was used to obtain the images. Contrast and brightness were adjusted to improve visualization but were kept constant across the different times and frames of the Z‐stack acquisition. Images correspond to Z‐stack compilations. Scale bars correspond to the indicated measures. (B) Comparison of microcolonies with large (> 10 μm) and small (< 5 μm) microcolonies shows significant differences in the mean GFP fluorescent intensity (Mann–Whitney *U* test, *p* < 10^−4^). (C) Correlation between microcolony area and fluorescence intensity of apoplastic *wbpL*::*GFP3* microcolonies indicates that higher expression is associated with smaller microcolonies. GFP, green fluorescent protein.

The analysis of expression of T3SS/Flagella within the context of apoplastic bacterial microcolonies showed a spatial distribution of phenotypic variants that support that Flagella^ON^/T3SS^OFF^ bacteria are somehow protected from the plant cell responses by Flagella^OFF^/T3SS^ON^ bacteria trans‐complementing their defense suppression defect (López‐Pagán et al. 2025). Thus, a possible explanation for the lack of viability differences between WbpL^ON^ and WbpL^OFF^ bacteria (Figure 3C) might be that these phenotypic variants may cooperate within the context of the microcolony, as shown for the T3SS and flagellar systems. A key aspect of the cooperative nature of the relationship between the different ON/OFF subpopulations of T3SS and flagellar genes is the spatial context of their respective expression within a growing microcolony (López‐Pagán et al. 2025). Thus, we investigated how *wbpL* expression is distributed within apoplastic microcolonies. To this purpose, we analyzed by confocal microscopy leaves inoculated with Pph *wbpL::GFP3* at 3 dpi (Figure 4A). Apoplastic microcolonies displayed a heterogeneous pattern of single‐cell expression very similar to the patterns described previously for flagella and the T3SS genes (López‐Pagán et al. 2025), compatible with stochastic switching of the *wbpL* locus during microcolony development. However, while flagella and the T3SS genes display a differential spatial distribution within the microcolony, with a preference for Flagella^OFF^/T3SS^ON^ bacteria in the proximal side of the microcolony, closest to the host cell surface, and an abundance of Flagella^ON^/T3SS^OFF^ in the more distal parts (López‐Pagán et al. 2025), no consistent zonal pattern was identified for expression of the *wbpL* locus (Figure 4A). Zones of the strongest fluorescent were sometimes visible in some microcolonies, but no reproducible common pattern for the localization of these areas was identified. Although we could not be ruled out that there might be a zonal pattern that may become apparent in, for example, different defense contexts, the simplest explanation for the available data is that such brighter areas as observed in our conditions may simply reflect denser areas within a given microcolony.

While no discernible zonal pattern was evident within each microcolony, one clear pattern did become apparent in these experiments when comparing the different microcolonies to each other, smaller microcolonies were consistently brighter, displaying stronger *wbpL::GFP3* fluorescence) (Figure 4A). Such association between size and overall gene expression intensity is not observed for the flagellar or T3SS genes, nor for constitutively expressed fluorescent reporter genes (López‐Pagán et al. 2025). To validate the observed trend, we first classified microcolonies into small or large (not considering intermediate sizes for this purpose) and measured the average GFP fluorescence of each microcolony by manually selecting the area of the microcolony before calculating the mean GFP fluorescence. The results validated the observed trend showing that mean GFP fluorescence is significantly higher in small colonies (Figure 4B). We also measured the areas and fluorescence of the microcolonies (all microcolonies, this time including intermediate sizes) and represented the one against the other (Figure 4C). The results obtained support the notion of a negative correlation between size and *wbpL::GFP* expression.

## 4 Discussion

The differences in *wbpL* expression observed when comparing apoplastic microcolonies have two interesting new aspects to consider. First, despite the heterogeneous ON/OFF switching there is a common trend for high or low *wbpL::GFP* expression (Figure 4A), which differs between microcolonies. Such a trend may support three possible scenarios: (i) differences in the microenvironments surrounding small versus large microcolonies may be causing a differential activation of *wbpL* expression within the entire microcolony, (ii) differences in expression of *wbpL* may be associated with the growth phase of the microcolony, with smaller microcolonies having started to grow later and being in a more active growth state that may require a higher rate to LPS synthesis, and (iii) expression of *wbpL* may be regulated by an epigenetic mechanism, like it happens with LPS genes for example in *Salmonella* (Cota et al. 2012), with the mean expression in each microcolony being a reflection of a common inherited status.

The second interesting aspect of these results is the negative correlation observed between the size of the microcolony and the intensity of *wbpL::GFP* fluorescence (Figure 4B,C). A typical *E. coli* cell possesses ∼2 × 10^6^ LPS molecules, covering about three‐quarters of the cell surface, with 70,000 molecules/min estimated to be exported to the OM, during active growth (reviewed in Whitfield et al. 2020). These numbers support the idea that the production of these macromolecules may require a substantial fraction of bacterial resources. Thus, higher production of WbpL might be associated with slower growth. Additionally, the potential impact of WbpL‐mediated changes to the LPS could determine that stronger expression of *wbpL::GFP* may lead to a stronger local defense restricting bacterial growth.

Phylogenetically, AtLORE is restricted to the Brasssicaceae family (Ranf et al. 2015), but analogous receptors might be present in other species, such as bean plants. In *Arabidopsis*, LPS triggers the biphasic production of reactive oxygen species (ROS), with a LORE‐dependent early ROS burst, and a late intracellular burst that is only partially dependent on LORE (Shang‐Guan et al. 2018). While the early ROS burst is not detected in *N. benthamiana*, tomato, barley or rice, a similar early ROS burst can be induced in soybean (Shang‐Guan et al. 2018). Interestingly, LPS‐triggered late ROS burst is only partially dependent on LORE in *Arabidopsis*, and is conserved in all the abovementioned plant species, suggesting the existence of an additional LPS perception system widely distributed in plants that mediates this second ROS response to LPS elicitation (Shang‐Guan et al. 2018). A comparative analysis of the LPS of a rough and smooth strain of Pph was performed by Gross et al. (1988), while LPS purification and partial characterization were achieved by Ramm et al. (2023), describing a rhamnose and fucose‐rich OPS. The OPS of many plant‐pathogenic bacteria comprise l‐rhamnose‐rich backbones, and synthetic oligorhamnans induce expression of pathogenesis‐related genes in *Arabidopsis* (Bedini et al. 2005). However, while this suggests that bacterial OPS might induce plant immunity, the ability to elicit plant immune responses by the purified OPS from Pph1448A, or any other natural OPS, has not yet been confirmed and thus remains a likely but untested possibility, also considering that plant receptors mediating OPS sensing have not yet been identified. In any case, in a scenario where stronger *wbpL* expression could lead to triggering stronger local plant defenses, these defenses would restrict bacterial growth without compromising bacterial viability, since PI staining shows no difference between WbpL^ON^ and WbpL^OFF^ bacteria (Figure 3C).

Future work will be necessary to establish the biological implications of the *wbpL* expression patterns described here, as well as to reveal the underlying mechanisms regulating the heterogeneous and bistable expression of this locus in this plant pathogen. But, the results presented here provide evidence of the conservation of an adaptive strategy between plant and animal pathogens: phenotypic heterogeneity of LPS genes. Interestingly, a very recent report has demonstrated through experimental observations and computational simulations, the adaptive value of the bistable expression of the LPS‐related *Salmonella opvAB* locus, regulated by epigenetic variation (Fernández‐Fernández et al. 2024). The report supports the notion of such variation as an evolutionary strategy for mutation avoidance in fluctuating environments, while providing experimental support to game theory models predicting that phenotypic heterogeneity is advantageous in changing and unpredictable environments. The findings from our group that crucial virulence determinants such as the T3SS (Rufián et al. 2016), flagella (López‐Pagán et al. 2025), and LPS (this report) display phenotypic heterogeneity in *P. syringae* during growth within the plant host, as these systems do in animal pathogens such as *Salmonella* (SPI1 T3SS; Bumann 2002; Hautefort et al. 2003; Saini et al. 2010; Flagella Freed et al. 2008 and LPS Cota et al. 2016) expands these evolutionary strategies to bacterial plant pathogens.

## Author Contributions

**Laura Mancera‐Miranda:** investigation, methodology, validation, visualization, writing – review and editing. **José S Rufián:** investigation, methodology, validation, visualization, writing – review and editing. **Nieves López‐Pagán:** investigation, methodology, validation, visualization, writing – review and editing. **Javier Ruiz‐Albert:** conceptualization, funding acquisition, writing – review and editing, methodology. **Carmen R Beuzón:** conceptualization, funding acquisition, writing – original draft.

## Conflicts of Interest

The authors declare no conflicts of interest.

## Supporting information

Figure S1 new.

Figure S2.

Figure S3.

supmat.

## Data Availability

The data that support the findings of this study are openly available in Flow Repository at http://flowrepository.org/id/FR-FCM-Z8X7, reference number R‐FCM‐Z8X7.

## References

[mbo370031-bib-0001] Appelmelk, B. J. , M. C. Martino , E. Veenhof , et al. 2000. “Phase Variation in H Type I and Lewis a Epitopes of *Helicobacter pylori* Lipopolysaccharide.” Infection and Immunity 68, no. 10: 5928–5932. 10992504 10.1128/iai.68.10.5928-5932.2000 PMC101556

[mbo370031-bib-0002] Bedini, E. , C. De Castro , G. Erbs , et al. 2005. “Structure‐Dependent Modulation of a Pathogen Response in Plants by Synthetic *O*‐Antigen Polysaccharides.” Journal of the American Chemical Society 127, no. 8: 2414–2416. 10.1021/ja0452166. 15724995

[mbo370031-bib-0003] Berry, M. C. , G. C. McGhee , Y. Zhao , and G. W. Sundin . 2009. “Effect of a *waaL* Mutation on Lipopolysaccharide Composition, Oxidative Stress Survival, and Virulence in *Erwinia amylovora* .” FEMS Microbiology Letters 291, no. 1: 80–87. 10.1111/j.1574-6968.2008.01438.x. 19076232

[mbo370031-bib-0004] Bertani, G. 1951. “Studies on Lysogenesis.” Journal of Bacteriology 62, no. 3: 293–300. 10.1128/jb.62.3.293-300.1951. 14888646 PMC386127

[mbo370031-bib-0005] Bogino, P. , M. Oliva , F. Sorroche , and W. Giordano . 2013. “The Role of Bacterial Biofilms and Surface Components in Plant‐Bacterial Associations.” International Journal of Molecular Sciences 14, no. 8: 15838–15859. 10.3390/ijms140815838. 23903045 PMC3759889

[mbo370031-bib-0006] Bretz, J. , L. Losada , K. Lisboa , and S. W. Hutcheson . 2002. “Lon Protease Functions as a Negative Regulator of Type III Protein Secretion in *Pseudomonas syringae* .” Molecular Microbiology 45: 397–409. 10.1046/j.1365-2958.2002.03008.x. 12123452

[mbo370031-bib-0007] Bumann, D. 2002. “Examination of *Salmonella* Gene Expression in an Infected Mammalian Host Using the Green Fluorescent Protein and Two‐Colour Flow Cytometry.” Molecular Microbiology 43, no. 5: 1269–1283. 11918812 10.1046/j.1365-2958.2002.02821.x

[mbo370031-bib-0008] Cota, I. , A. B. Blanc‐Potard , and J. Casadesús . 2012. “STM2209‐STM2208 (opvAB): A Phase Variation Locus of *Salmonella enterica* Involved in Control of *O*‐Antigen Chain Length.” PLoS ONE 7, no. 5: e36863. 10.1371/journal.pone.0036863. 22606300 PMC3350482

[mbo370031-bib-0009] Cota, I. , B. Bunk , C. Spröer , J. Overmann , C. König , and J. Casadesús . 2016. “OxyR‐Dependent Formation of DNA Methylation Patterns in OpvAB OFF and OpvAB ON Cell Lineages of *Salmonella enterica* .” Nucleic Acids Research 44, no. 8: 3595–3609. 26687718 10.1093/nar/gkv1483 PMC4856963

[mbo370031-bib-0010] Cuppels, D. A. 1986. “Generation and Characterization of Tn5 Insertion Mutations in *Pseudomonas syringae* pv. *tomato* .” Applied and Environmental Microbiology 51, no. 2: 323–327. 16346988 10.1128/aem.51.2.323-327.1986 PMC238867

[mbo370031-bib-0011] Drigues, P. , D. Demery‐Lafforgue , A. Trigalet , P. Dupin , D. Samain , and J. Asselineau . 1985. “Comparative Studies of Lipopolysaccharide and Exopolysaccharide From a Virulent Strain of *Pseudomonas solanacearum* and From Three Avirulent Mutants.” Journal of Bacteriology 162, no. 2: 504–509. https://journals.asm.org/journal/jb. 3988700 10.1128/jb.162.2.504-509.1985 PMC218876

[mbo370031-bib-0012] Fernández‐Fernández, R. , D. R. Olivenza , E. Weyer , A. Singh , J. Casadesús , and M. A. Sánchez‐Romero . 2024. “Evolution of a Bistable Genetic System in Fluctuating and Nonfluctuating Environments.” Proceedings of the National Academy of Sciences of the United States of America 121, no. 36: 2322371121. 10.1073/pnas.2322371121. PMC11388349 39213178

[mbo370031-bib-0013] Freed, N. E. , O. K. Silander , B. Stecher , A. Böhm , W. D. Hardt , and M. Ackermann . 2008. “A Simple Screen to Identify Promoters Conferring High Levels of Phenotypic Noise.” PLoS Genetics 4, no. 12: e1000307. 10.1371/journal.pgen.1000307. 19096504 PMC2588653

[mbo370031-bib-0014] Funahara, Y. , and H. Nikaido . 1980. “Asymmetric Localization of Lipopolysaccharides on the Outer Membrane of *Salmonella typhimurium* .” Journal of Bacteriology 141, no. 3: 1463–1465. 6988417 10.1128/jb.141.3.1463-1465.1980 PMC293861

[mbo370031-bib-0015] Goldman, R. C. , and L. Leive . 1980. “Heterogeneity of Antigenic‐Side‐Chain Length in Lipopolysaccharide From *Escherichia coli* 0111 and *Salmonella typhimurium* LT2.” European Journal of Biochemistry 107, no. 1: 145–153. 10.1111/j.1432-1033.1980.tb04635.x. 6156828

[mbo370031-bib-0054] Gross, M. , H. Mayer , C. Widemann , et al. 1988. “Comparative Analysis of the Lipopolysaccharides of a Rough and a Smooth Strain of *Pseudomonas syringae* pv. *phaseolicola* .” Archives of Microbiology 149: 372–376. 10.1007/BF00411658.

[mbo370031-bib-0017] Hautefort, I. , M. Proença , and J. C. D. Hinton . 2003. “Single‐Copy Green Fluorescent Protein Gene Fusions Allow Accurate Measurement of *Salmonella* Gene Expression In Vitro and During Infection of Mammalian Cells.” Applied and Environmental Microbiology 69, no. 12: 7480–7491. 10.1128/AEM.69.12.7480-7491.2003. 14660401 PMC310007

[mbo370031-bib-0018] Herskowitz, I. , and D. Hagen . 1980. “The Lysis‐Lysogeny Decision of Phage λ: Explicit Programming and Responsiveness.” Annual Review of Genetics 14: 399–445. www.annualreviews.org. 10.1146/annurev.ge.14.120180.002151 6452089

[mbo370031-bib-0019] Holst, O. , and H. Brade . 1992. “Chemical Structure of the Core Region of Lipopolysaccharides.” In Bacterial Endotoxic Lipopolysaccharides, edited by D. C. Morrison and J. L. Ryan , 171–205. CRC Press.

[mbo370031-bib-0020] Huynh, T. V. , D. Dahlbeck , and B. J. Staskawicz . 1989. “Bacterial Blight of Soybean: Regulation of a Pathogen Gene Determining Host Cultivar Specificity.” Science 245, no. 4924: 1374–1377. 10.1126/science.2781284. 2781284

[mbo370031-bib-0021] Kutschera, A. , U. Schombel , M. Wröbel , N. Gisch , and S. Ranf . 2019. “Loss of *wbpL* Disrupts *O*‐Polysaccharide Synthesis and Impairs Virulence of Plant‐Associated *Pseudomonas* Strains.” Molecular Plant Pathology 20, no. 11: 1535–1549. 10.1111/mpp.12864. 31559681 PMC6804347

[mbo370031-bib-0022] Lehtinen, J. , J. Nuutila , and E. M. Lilius . 2004. “Green Fluorescent Protein‐Propidium Iodide (GFP‐PI) Based Assay for Flow Cytometric Measurement of Bacterial Viability.” Cytometry, Part A 60, no. 2: 165–172. 10.1002/cyto.a.20026. 15290717

[mbo370031-bib-0023] Lerouge, I. , and J. Vanderleyden . 2002. “ *O*‐Antigen Structural Variation: Mechanisms and Possible Roles in Animal/Plant–Microbe Interactions.” FEMS Microbiology Reviews 26, no. 1: 17–47. 10.1111/j.1574-6976.2002.tb00597.x. 12007641

[mbo370031-bib-0024] Li, C. H. , K. C. Wang , Y. H. Hong , et al. 2014. “Roles of Different Forms of Lipopolysaccharides in *Ralstonia solanacearum* Pathogenesis.” Molecular Plant—Microbe Interactions 27, no. 5: 471–478. 10.1094/MPMI-08-13-0248-R. 24580105

[mbo370031-bib-0025] Long, C. M. , P. A. Beare , D. Cockrell , et al. 2024. “Natural Reversion Promotes LPS Elongation in an Attenuated *Coxiella burnetii* Strain.” Nature Communications 15, no. 1: 1–8. 10.1038/s41467-023-43972-y. PMC10808227 38267444

[mbo370031-bib-0026] López‐Pagán, N. , J. S. Rufián , J. Luneau , et al. 2025. “ *Pseudomonas syringae* Subpopulations Cooperate by Coordinating Flagellar and Type III Secretion Spatiotemporal Dynamics to Facilitate Plant Infection.” Nature Microbiology 10: 958–972. 10.1038/s41564-025-01966-0. PMC11964935 40175722

[mbo370031-bib-0027] Mlynek, K. D. , C. T. Lopez , D. P. Fetterer , J. A. Williams , and J. A. Bozue . 2022. “Phase Variation of LPS and Capsule Is Responsible for Stochastic Biofilm Formation in *Francisella tularensis* .” Frontiers in Cellular and Infection Microbiology 11: 808550. 10.3389/fcimb.2021.808550. 35096655 PMC8795689

[mbo370031-bib-0028] Mostowy, R. J. , and K. E. Holt . 2018. “Diversity‐Generating Machines: Genetics of Bacterial Sugar‐Coating.” In Trends in Microbiology: 1008–1021. 10.1016/j.tim.2018.06.006. 30037568 PMC6249986

[mbo370031-bib-0029] Novick, A. , and M. Weiner . 1957. “Enzyme Induction as an All‐or‐None Phenomenon.” Proceedings of the National Academy of Sciences 43: 553–566. https://www.pnas.org. 10.1073/pnas.43.7.553 PMC528498 16590055

[mbo370031-bib-0030] O'Leary, B. M. , H. C. Neale , C. Geilfus , R. W. Jackson , D. L. Arnold , and G. M. Preston . 2016. “Early Changes in Apoplast Composition Associated With Defence and Disease in Interactions Between *Phaseolus vulgaris* and the Halo Blight Pathogen *Pseudomonas syringae* pv. *phaseolicola* .” Plant, Cell & Environment 39, no. 10: 2172–2184. 10.1111/pce.12770. PMC5026161 27239727

[mbo370031-bib-0031] Omaleki, L. , P. J. Blackall , T. Cuddihy , et al. 2022. “Phase Variation in the Glycosyltransferase Genes of *Pasteurella multocida* Associated With Outbreaks of Fowl Cholera on Free‐Range Layer Farms.” Microbial Genomics 8, no. 3: 1–15. 10.1099/mgen.0.000772. PMC9176279 35266868

[mbo370031-bib-0032] Patel, R. R. , P. P. Kandel , E. Traverso , K. L. Hockett , and L. R. Triplett . 2021. “ *Pseudomonas syringae* pv. *phaseolicola* Uses Distinct Modes of Stationary‐Phase Persistence to Survive Bacteriocin and Streptomycin Treatments.” mBio 12, no. 2: 1–17. 10.1128/mBio. PMC8092213 33849974

[mbo370031-bib-0033] Petrocelli, S. , M. L. Tondo , L. D. Daurelio , and E. G. Orellano . 2012. “Modifications of *Xanthomonas axonopodis* pv. *citri* Lipopolysaccharide Affect the Basal Response and the Virulence Process During Citrus Canker.” PLoS ONE 7, no. 7: e40051. 10.1371/journal.pone.0040051. 22792211 PMC3391215

[mbo370031-bib-0034] Rahme, L. G. , M. N. Mindrinos , and N. J. Panopoulos . 1992. “Plant and Environmental Sensory Signals Control the Expression of *hrp* Genes in *Pseudomonas syringae* pv. *phaseolicola* .” Journal of Bacteriology 174: 3499–3507. 10.1128/jb.174.11.3499-3507.1992. 1592805 PMC206034

[mbo370031-bib-0055] Ramm, M. , M. Lobe , and M. Hamburger . 2003. “A Simple Method for Preparation of D‐rhamnose.” Carbohydrate Research 338: 109–112. 10.1016/s0008-6215(02)00353-1. 12504387

[mbo370031-bib-0035] Ranf, S. 2016. “Immune Sensing of Lipopolysaccharide in Plants and Animals: Same But Different.” PLoS Pathogens 12, no. 6: 1–7. 10.1371/journal.ppat.1005596. PMC4900518 27281177

[mbo370031-bib-0036] Ranf, S. , N. Gisch , M. Schäffer , et al. 2015. “A Lectin S‐Domain Receptor Kinase Mediates Lipopolysaccharide Sensing in *Arabidopsis thaliana* .” Nature Immunology 16: 426–433. 10.1038/ni.3124. 25729922

[mbo370031-bib-0037] Rapicavoli, J. N. , B. Blanco‐Ulate , A. Muszyński , et al. 2018. “Lipopolysaccharide *O*‐Antigen Delays Plant Innate Immune Recognition of *Xylella fastidiosa* .” Nature Communications 9, no. 1: 390. 10.1038/s41467-018-02861-5. PMC5786101 29374171

[mbo370031-bib-0038] Rudolph, K. W. W. , M. Gross , M. Neugebauer , et al. 1989. “Extracellular Polysaccharides as Determinants of Leaf Spot Diseases Caused by *Pseudomonads* and *Xanthomonad* .” In Phytotoxins and Plan Pathogenesis, edited by A. Graniti , R. D. Durbin and A. Ballio , 177–218. Springer.

[mbo370031-bib-0039] Rufián, J. S. , D. López‐Márquez , N. López‐Pagán , M. Grant , J. Ruiz‐Albert , and C. R. Beuzón . 2018. “Generating Chromosome‐Located Transcriptional Fusions to Fluorescent Proteins for Single‐Cell Gene Expression Analysis in *Pseudomonas syringae* .” In Host–Pathogen Interactions: Methods and Protocols, edited by C. Medina and F. J. López‐Baena , 183–199. Springer New York. 10.1007/978-1-4939-7604-1_15. 29288455

[mbo370031-bib-0040] Rufián, J. S. , N. López‐Pagán , J. Ruiz‐Albert , and C. R. Beuzón . 2022. “Single‐Cell Analysis of the Expression of *Pseudomonas syringae* Genes Within the Plant Tissue.” Journal of Visualized Experiments no. 188: e64614. 10.3791/64614. 36282707

[mbo370031-bib-0041] Rufián, J. S. , M. A. Sánchez‐Romero , D. López‐Márquez , et al. 2016. “ *Pseudomonas syringae* Differentiates into Phenotypically Distinct Subpopulations During Colonization of a Plant Host.” Environmental Microbiology 18, no. 10: 3593–3605. 10.1111/1462-2920.13497. 27516206

[mbo370031-bib-0042] Saini, S. , J. R. Ellermeier , J. M. Slauch , and C. V. Rao . 2010. “The Role of Coupled Positive Feedback in the Expression of the SPI1 Type Three Secretion System in *Salmonella’* .” PLoS Pathogens 6, no. 7: e1001025. 10.1371/journal.ppat.1001025. 20686667 PMC2912647

[mbo370031-bib-0043] Schoonejans, E. , D. Expert , and A. Toussaint . 1987. “Characterization and Virulence Properties of *Erwinia chrysanthemi* Lipopolysaccharide‐Defective, Phi EC2‐Resistant Mutants.” Journal of Bacteriology 169, no. 9: 4011–4017. https://journals.asm.org/journal/jb. 3624200 10.1128/jb.169.9.4011-4017.1987 PMC213701

[mbo370031-bib-0044] Shang‐Guan, K. , M. Wang , N. M. P. S. Htwe , et al. 2018. “Lipopolysaccharides Trigger Two Successive Bursts of Reactive Oxygen Species at Distinct Cellular Locations.” Plant Physiology 176, no. 3: 2543–2556. 10.1104/pp.17.01637. 29431629 PMC5841728

[mbo370031-bib-0045] Sijmons, D. , A. J. Guy , A. K. Walduck , and P. A. Ramsland . 2022. “ *Helicobacter pylori* and the Role of Lipopolysaccharide Variation in Innate Immune Evasion.” Frontiers in Immunology 13: 1–13. 10.3389/fimmu.2022.868225. PMC9136243 35634347

[mbo370031-bib-0046] Silhavy, T. J. , D. Kahne , and S. Walker . 2010. “The Bacterial Cell Envelope.” Cold Spring Harbor Perspectives in Biology 2, no. 5: a000414. 10.1101/cshperspect.a000414. 20452953 PMC2857177

[mbo370031-bib-0047] Taira, S. , J. Tuimala , E. Roine , E. L. Nurmiaho‐Lassila , H. Savilahti , and M. Romantschuk . 1999. “Mutational Analysis of the *Pseudomonas syringae* pv. *tomato hrpA* Gene Encoding Hrp Pilus Subunit.” Molecular Microbiology 34: 737–744. 10.1046/j.1365-2958.1999.01635.x. 10564513

[mbo370031-bib-0048] Teverson, D. M. 1991. “Genetics of Pathogenicity and Resistance in the Halo‐Blight Disease of Beans in Africa.” PhD thesis, University of Birmingham.

[mbo370031-bib-0049] Thwaites, R. , P. D. Spanu , N. J. Panopoulos , C. Stevens , and J. W. Mansfield . 2004. “Transcriptional Regulation of Components of the Type III Secretion System and Effectors in *Pseudomonas syringae* pv. *phaseolicola* .” Molecular Plant–Microbe Interactions: MPMI 17: 1250–1258. 10.1094/MPMI.2004.17.11.1250. 15553250

[mbo370031-bib-0050] van der Woude, M. W. 2011. “Phase Variation: How to Create and Coordinate Population Diversity.” Current Opinion in Microbiology 14, no. 2: 205–211. 10.1016/j.mib.2011.01.002. 21292543

[mbo370031-bib-0051] Weiser, J. N. , D. J. Maskell , P. D. Butler , A. A. Lindberg , and E. R. Moxon . 1990. “Characterization of Repetitive Sequences Controlling Phase Variation of *Haemophilus influenzae* Lipopolysaccharide.” Journal of Bacteriology 172, no. 6: 3304–3309. https://journals.asm.org/journal/jb. 1693145 10.1128/jb.172.6.3304-3309.1990 PMC209140

[mbo370031-bib-0052] Whitfield, C. , D. M. Williams , and S. D. Kelly . 2020. “Lipopolysaccharide *O*‐Antigens‐Bacterial Glycans Made to Measure.” Journal of Biological Chemistry 295, no. 31: 10593–10609. 10.1074/jbc.REV120.009402. 32424042 PMC7397119

[mbo370031-bib-0053] Xiao, Y. , Y. Lu , S. Heu , and S. W. Hutcheson . 1992. “Organization and Environmental Regulation of the *Pseudomonas syringae* pv. *syringae* 61 *hrp* Cluster.” Journal of Bacteriology 174: 1734–1741. 10.1128/jb.174.6.1734-1741.1992. 1548225 PMC205773

