# Supplementary figures and images for: Pseudomonas syringae Lipopolysaccharide Synthesis Gene wbpL Displays Heterogeneous Expression Within In Vitro and In Planta Populations
Source: Microbiologyopen. 2025 Jul 22;14(4):e70031. doi: 10.1002/mbo3.70031 (PMC12280811; doi:10.1002/mbo3.70031)

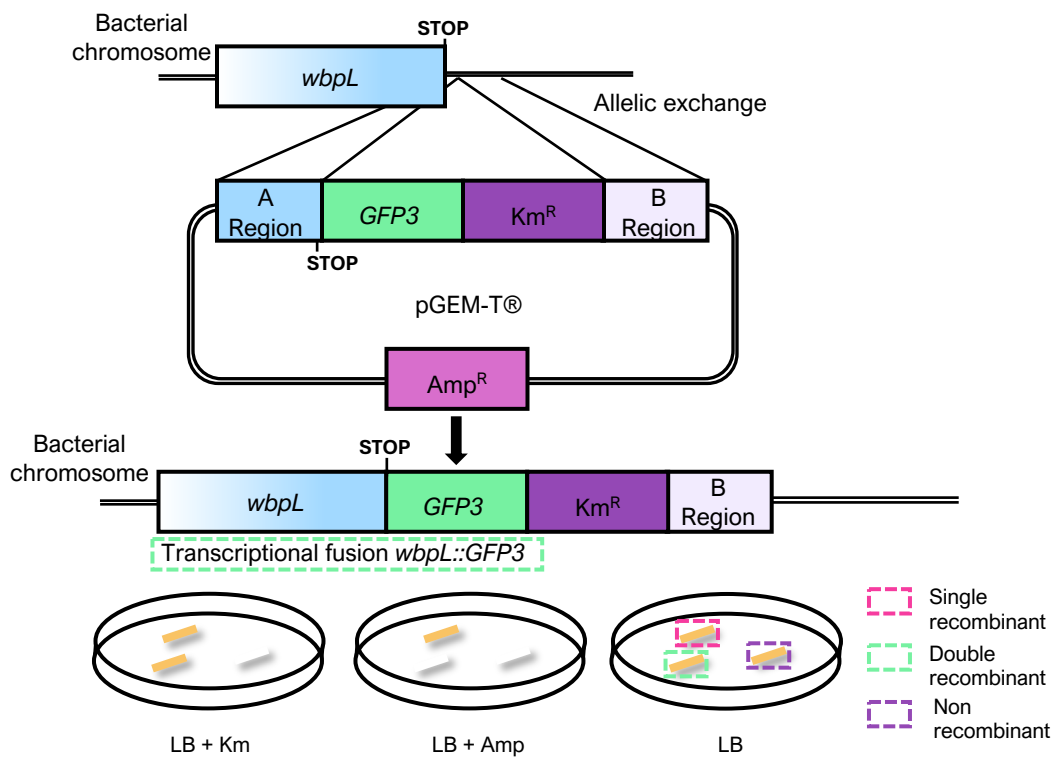

Supplement: Supplementary file 1 — Figure S1 new. [file MBO3-14-e70031-s004.pdf]

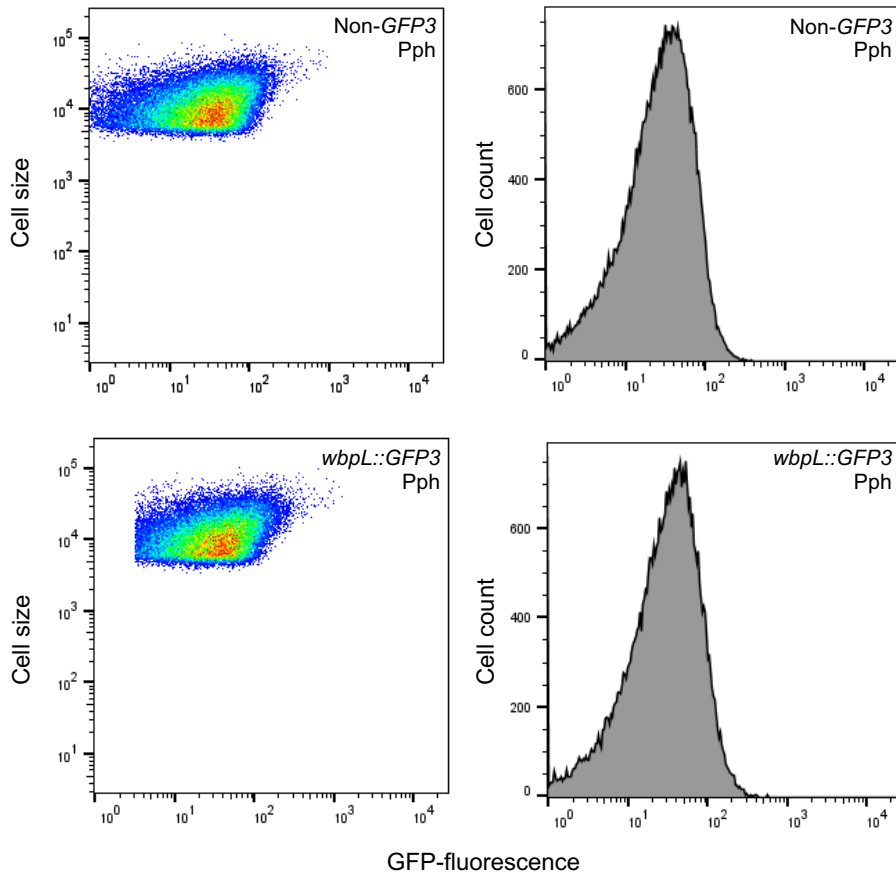

## RCV

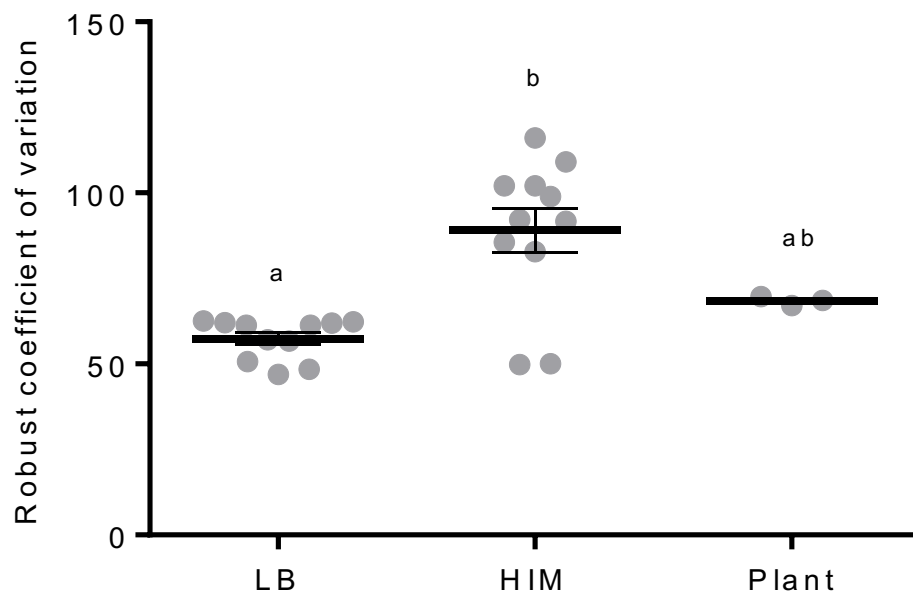

Supplement: Supplementary file 2 — Figure S2. [file MBO3-14-e70031-s003.pdf]

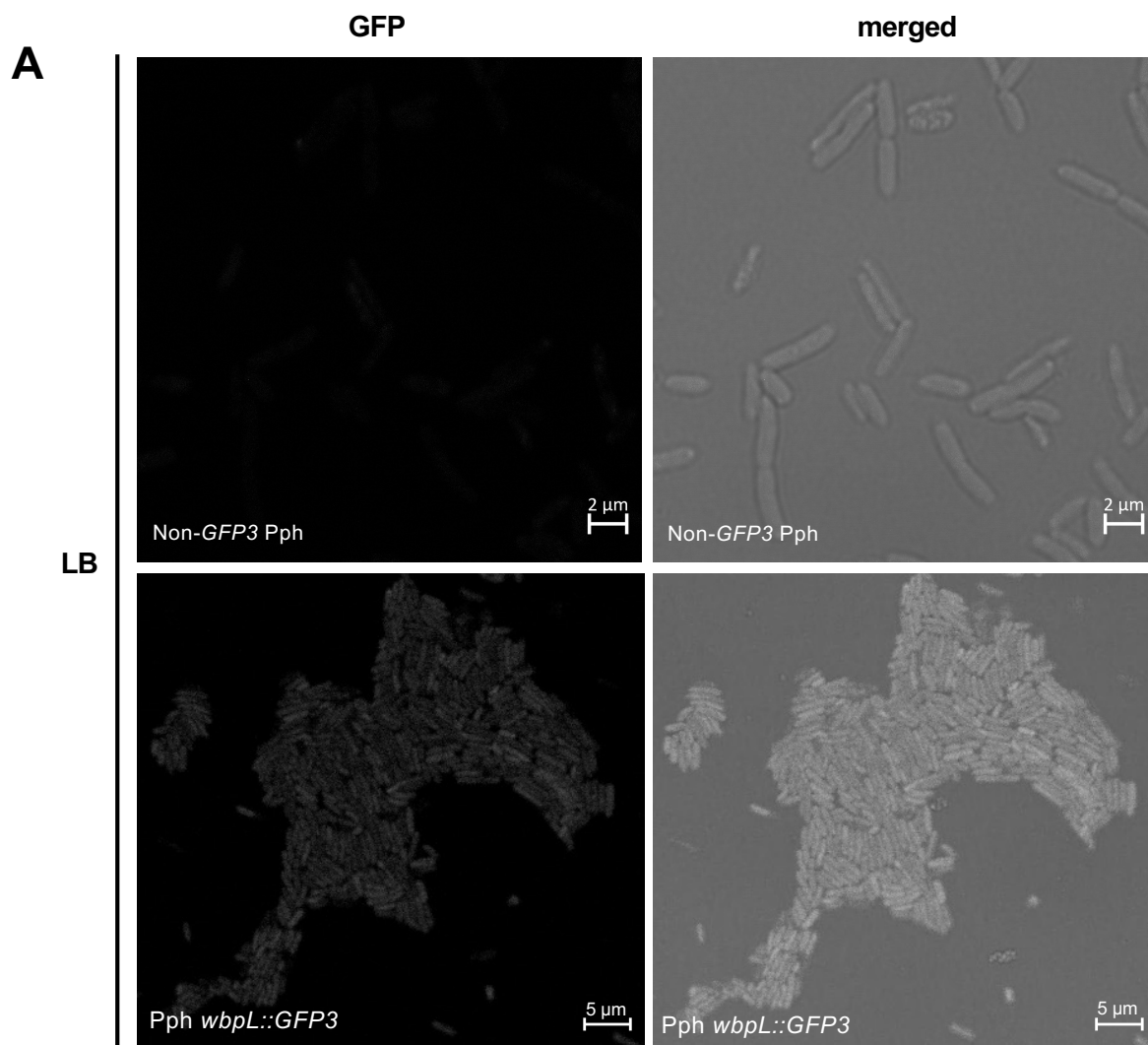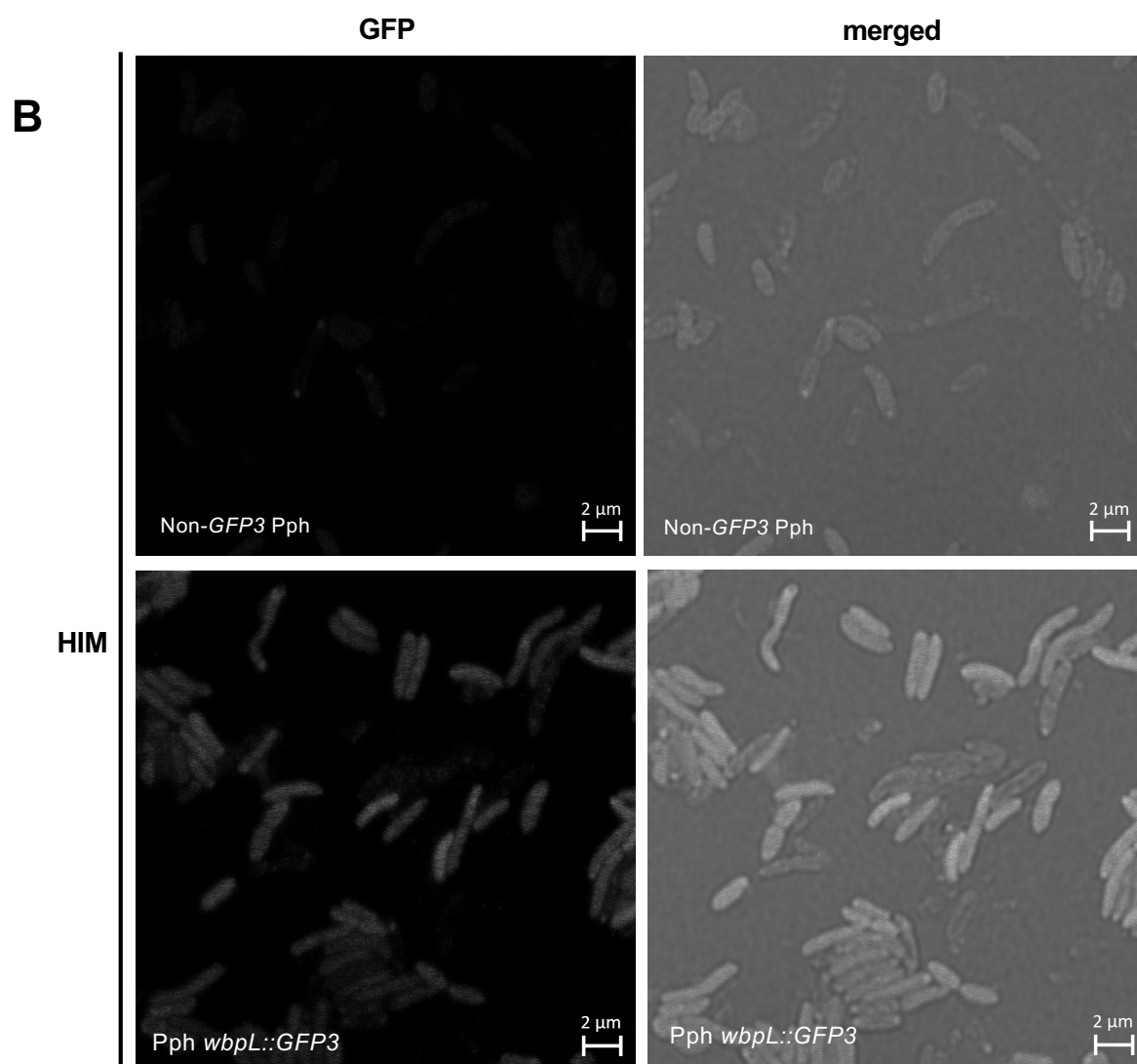

Supplement: Supplementary file 3 — Figure S3. [file MBO3-14-e70031-s002.pdf]
