# Supplementary material for: Pseudomonas syringae Lipopolysaccharide Synthesis Gene wbpL Displays Heterogeneous Expression Within In Vitro and In Planta Populations
Source: Microbiologyopen. 2025 Jul 22;14(4):e70031. doi: 10.1002/mbo3.70031 (PMC12280811; doi:10.1002/mbo3.70031)
Supplement: Supplementary file 4 — supmat. [file MBO3-14-e70031-s001.docx]

**Figure S1**. **Schematic representation of the process used to introduce and select for the allelic exchange into the chromosome of a transcriptional fusion of the gene *wbpL* to *GFP3* (*wbpL::GFP3*)*.*** A plasmid containing the *GFP3*-Km^R^ cassette flanked by two 500 nucleotide regions (A and B), respectively corresponding to the 500▒bp upstream and downstream the STOP codon of *wbpL*, was transformed into *P. syringae* (Pto or Pph). The *GFP3* section of the cassette (in green) includes the *GFP3* ORF preceded by its own ribosomal binding site (rbs) but lacking a promoter region, thus *GFP3* transcription depends on the *wbpL* promoter, while *GFP3* translation depends on its own rbs, which results in the expression of a single mRNA but two separate proteins (WbpL and GFP3). The cassette carries a kanamycin resistance gene (Km^R^ in purple) with its own constitutive promoter and ribosomal binding site (RBS) to allow selection of transformed clones carrying an integration of the *wbpL::GFP3* allele using antibiotic selection. Integration of the plasmid within the chromosome by a single recombination event renders bacteria Amp and Km resistant, whereas a double recombination event integrating the *GFP3*-Km cassette downstream *wbpL* by allelic exchange, generating the transcriptional fusion *wbpL::GFP3,* would render Km resistant, Amp sensitive bacteria. Thus, replica plating in LB+Km, LB+Amp and LB allows identification of double recombinants carrying the fusion but not the plasmid integration.

**Figure S2**. **Phenotypic heterogeneity of *wbpL* is maintained at different growth stages and is larger among HIM-grown bacteria.** Flow-cytometry analysis of a Pph derivative strain carrying a chromosome-located *wbpL*:*:GFP3* (upper panels), in early exponential growth in LB is shown as dot plots representing GFP fluorescence intensity *versus* cell size. Data are represented as arbitrary units in logarithmic scale. Histograms show GFP fluorescence *versus* cell count for the same data. Data displayed corresponds to that collected for at least 100,000 events per sample. The non-GFP graphs shows autofluorescence levels displayed by the Pph reference strain not carrying any fluorescent gene marker. Results obtained are comparable to those obtained at stationary growth phase and shown in Figure 1A. Graph (lowest panel) shows the robust coefficient of variation (RCV) calculate for all replicates and FC experiments carried out for Pph 1448▒A *wbpL::GFP3* expression *in vitro* and *in planta* (as indicated). Results marked with different letters indicate those stablished as significantly different by ANOVA (P▒>▒0.0001).

**Figure S3**. Confocal microscopic images of LB-grown (**A**) or HIM-grown (**B**) of non-GFP3 Pph reference strain versus Pph *wbpL::GFP3*. Microscopy images show in the GFP channel the fluorescence of GFP (in white) as reporter of wbpL gene expression. Bright field is used to visualize all bacteria regardless of wbpL expression (merged). Scale bars correspond to the values indicated. Contrast and brightness were adjusted to improve visualization but were kept constant across the different conditions and channels.
